# Global Trends in Research on Social Media and Cosmetic Surgery Consideration: A Bibliometric Analysis

**DOI:** 10.1007/s00266-026-05687-5

**Published:** 2026-02-12

**Authors:** Haoyue Wang, Mumtaz Aini Binti Alivi, Siti Ezaleila Binti Mustafa, Jiaqing Xu, Nasrullah Dharejo

**Affiliations:** Faculty of Arts and Social Science, University of Malay, 50603 Kuala Lumpur, Wilayah Persekutuan Malaysia

**Keywords:** Bibliometric analysis, Social media, Cosmetic surgery, Research trends, International collaboration, Citation analysis

## Abstract

**Background:**

Scholarly interest in the relationship between social media and cosmetic surgery has increased substantially, as digital platforms play an increasingly central role in shaping aesthetic norms, body image perceptions, and elective medical decision-making. However, the rapid expansion of this literature has yet to be systematically mapped at a global level.

**Objective:**

This study aims to provide a comprehensive bibliometric overview of research examining the relationship between social media and cosmetic surgery, with particular attention to publication trends, knowledge structures, collaborative patterns, and thematic evolution over time.

**Methods:**

A systematic bibliometric analysis was conducted on 525 publications retrieved from the Scopus and Web of Science databases, covering the period from 2010 to 2024. Studies were identified following the PRISMA 2020 framework and analyzed using VOSviewer to visualize annual publication trends, co-authorship networks, co-citation structures, and keyword co-occurrence clusters.

**Results:**

Findings indicate an exponential rise in research activity post-2020, with the USA leading in publication output yet exhibiting relatively low international collaboration. Citation analysis highlights several seminal contributions that have shaped the field's clinical, psychological, and sociocultural dimensions. Thematic mapping reveals a notable shift from platform-specific studies (e.g., Facebook, Instagram) toward emerging concerns such as ethnic diversity, body dissatisfaction, medical ethics, and digital patient education. Furthermore, keyword trends suggest increasing academic attention to the psychosocial implications of algorithmic beauty standards.

**Conclusions:**

This bibliometric review demonstrates the rapid growth and increasing interdisciplinarity of scholarship on social media and cosmetic surgery, while also highlighting structural imbalances in global knowledge production. The findings clarify the intellectual landscape of the field and identify emerging research directions to inform future theoretical development and empirical inquiry.

**Level of Evidence V:**

This journal requires that authors assign a level of evidence to each article. For a full description of these Evidence-Based Medicine ratings, please refer to the Table of Contents or the online Instructions to Authors  www.springer.com/00266.

## Introduction

The intersection of social media usage and cosmetic surgery consideration has emerged as a significant area of study in recent years, reflecting the growing influence of digital platforms on body image perception and aesthetic choices [18]. In the age of algorithmic media and visual self-presentation, the body has become increasingly subjected to digital visibility, aesthetic norms, and constant networked comparison [3]. Platforms such as Instagram, TikTok, and Snapchat do not merely reflect existing beauty ideals, they actively construct and amplify them through algorithmically promoted content, augmented filters, and influencer-driven narratives [16]. These platforms foster a *“hyperreal”* body culture, where the lines between natural appearance and digitally enhanced aesthetics are progressively blurred. Within these digital environments, users are continuously exposed to filtered images, beauty-centric content, and conversations surrounding cosmetic procedures, all of which may shape their perceptions of ideal beauty and influence their decisions regarding cosmetic interventions [18]. In particular, platforms like Instagram, Snapchat, and TikTok promote idealized images through filters, algorithm-driven feeds, and influencer culture, which may reinforce narrow beauty norms and trigger appearance-based anxiety [8]. The phenomenon, often termed "Snapchat dysmorphia" or "Instagram face," has caught the attention of researchers across multiple disciplines, from psychology and sociology to plastic surgery and digital communication [17, 21]. From a social comparison theory perspective [13], such platforms provide constant visual stimuli for users to compare themselves with unattainable ideals, potentially leading to dissatisfaction, self-objectification, and the pursuit of appearance-enhancing interventions.

Moreover, the global cosmetic surgery industry has expanded dramatically in parallel with the cultural and technological rise of social media. Recent data from the International Society of Aesthetic Plastic Surgery [2] highlight this upward trajectory, with a growing number of patients explicitly citing social media exposure as a motivating factor in their decisions to pursue elective aesthetic procedures [11]. This phenomenon underscores not only a commercial boom, but also the increasing integration of digital beauty culture into personal medical choices. As user-generated content, influencer endorsements, and algorithmically tailored feeds continue to normalize aesthetic enhancement, the boundaries between personal agency and media influence become progressively blurred [15]. These developments have catalyzed a wide spectrum of scholarly inquiry, ranging from psychological impacts, such as body dissatisfaction and social comparison, to marketing strategies, informed consent, and medical ethics [23]. However, as the cosmetic surgery industry aligns more closely with platform-based beauty ideals, new concerns emerge regarding the role of social media in potentially shaping, or even distorting, consumer perceptions and clinical expectations.

Despite the exponential growth of scholarly work on the relationship between social media use and cosmetic surgery, the field still lacks a comprehensive bibliometric synthesis that systematically maps its intellectual structure, conceptual evolution, and global research dynamics. Existing studies have predominantly focused on empirical associations or theoretical frameworks, leaving the broader research landscape underexplored in terms of publication trends, influential contributors, and thematic development [5, 22, 25]. Bibliometric analysis, as a robust quantitative method, not only facilitates the detection of emerging research frontiers, but also reveals the intellectual foundations and collaborative structures that have shaped the field's development [29]. Accordingly, this study provides a systematic bibliometric analysis of scholarly literature at the intersection of social media and cosmetic surgery. Drawing on publications indexed in both the Scopus and Web of Science databases between 2010 and 2024 and applying the PRISMA 2020 framework alongside VOS viewer-based network visualization, the study aims to map the intellectual landscape of this field by identifying key research clusters, influential authors, institutional contributions, and evolving thematic trends.

## Methodology

This study adopted a systematic bibliometric approach guided by the PRISMA 2020 statement to examine the scientific landscape concerning the influence of social media on cosmetic surgery [20]. This methodology ensured transparent, comprehensive, and replicable literature retrieval, screening, and analysis.

### Searching Strategy

The WoS and Scopus databases were selected for their extensive coverage of peer-reviewed scientific literature and robust indexing capabilities. The search strategy incorporated relevant keywords connected by Boolean operators, focusing on terms related to social media platforms and cosmetic surgery: (("social media" OR "social network*" OR "Facebook" OR "Instagram" OR "TikTok" OR "snapchat") AND ("cosmetic surgery" OR "aesthetic surgery" OR "plastic surgery" OR "cosmetic procedure*")).

### Eligibility Criteria and Data Extraction

**Fig. 1:**
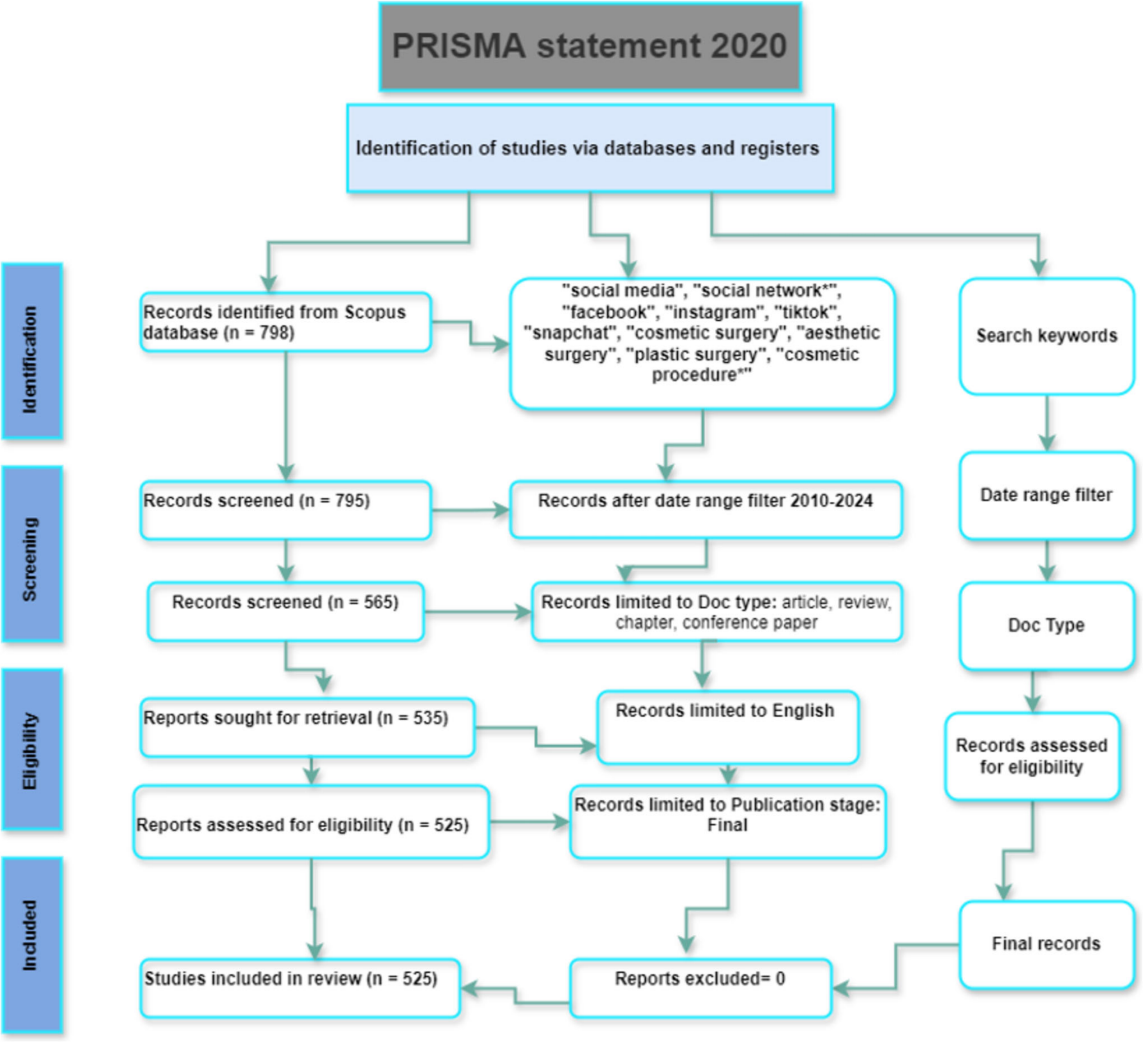
PRISMA template for exclusion and inclusion criteria

Following PRISMA 2020 guidelines, we implemented specific inclusion and exclusion criteria. Publications were included if they were published between 2010 and 2024, written in English, and in their final publication stage. Eligible document types encompass research articles, reviews, book chapters, and conference papers focusing on social media and cosmetic surgery. We excluded pre-2010 publications, non-English works, pre-prints, editorials, letters, notes, and conference abstracts without full text. As shown in Fig. 1, a total of 798 records were initially retrieved from the database. After limiting the timeframe to 2010–2024**,** 795 records remained. Subsequent filtering by document type (research articles, reviews, book chapters, and full conference papers) reduced the sample to 565. Limiting to English-language publications yielded 535, and final-stage publication filtering resulted in 525 documents included in the final analysis.

### Data Analysis Framework

VOS viewer software facilitated multidimensional bibliometric examination of the final dataset. The analysis framework included:

- Annual publication trend analysis
- Country collaboration analysis
- Institutional collaboration analysis
- Author co-authorship analysis
- Keyword co-occurrence analysis
- Citation analysis

This multi-dimensional approach enabled the identification of publication dynamics, leading contributors, global collaboration patterns, thematic developments, and foundational literature. The methodology provides a replicable and data-driven basis for understanding the structure, evolution, and emerging directions of scholarly inquiry at the intersection of social media and cosmetic surgery.

## Results

### Publication Trend Over Time

**Fig. 2:**
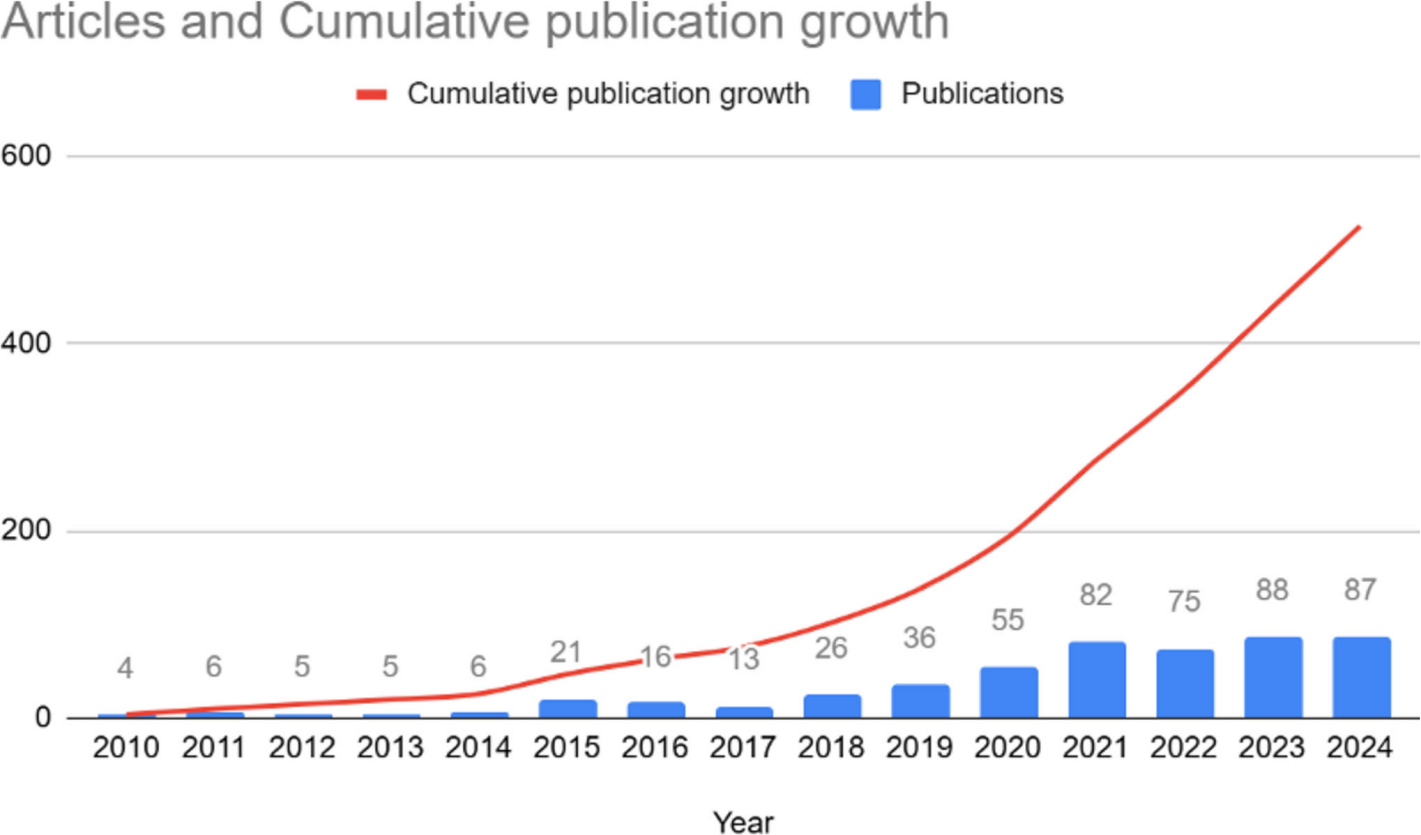
Publication output

The annual publication analysis reveals a marked upward trajectory in research on the intersection between social media and cosmetic surgery from 2010 to 2024. During the early stage (2010–2014), scholarly output remained modest, averaging 4–6 publications per year. A noticeable increase occurred in 2015, with the number of publications rising to 21, indicating the field’s emerging visibility. A phase of accelerated growth began in 2020, with annual publication counts consistently exceeding 75 articles. The field reached its peak output in 2021, recording 82 publications, and maintained a high volume through subsequent years, ranging between 75 and 88 publications annually up to 2024. This pattern, characterized by both cumulative and annual growth (see Fig. 2), demonstrates the evolution of the field from a niche research topic to a recognized scholarly domain. The exponential increase in output since 2019 reflects growing academic interest and the topic’s alignment with broader public health, psychological, and digital media discourses [28]. The sustained high productivity in recent years underscores the field’s maturation, interdisciplinary relevance, and continued academic significance in contemporary healthcare and communication research.

### Most Relevant Journals

**Fig. 3:**
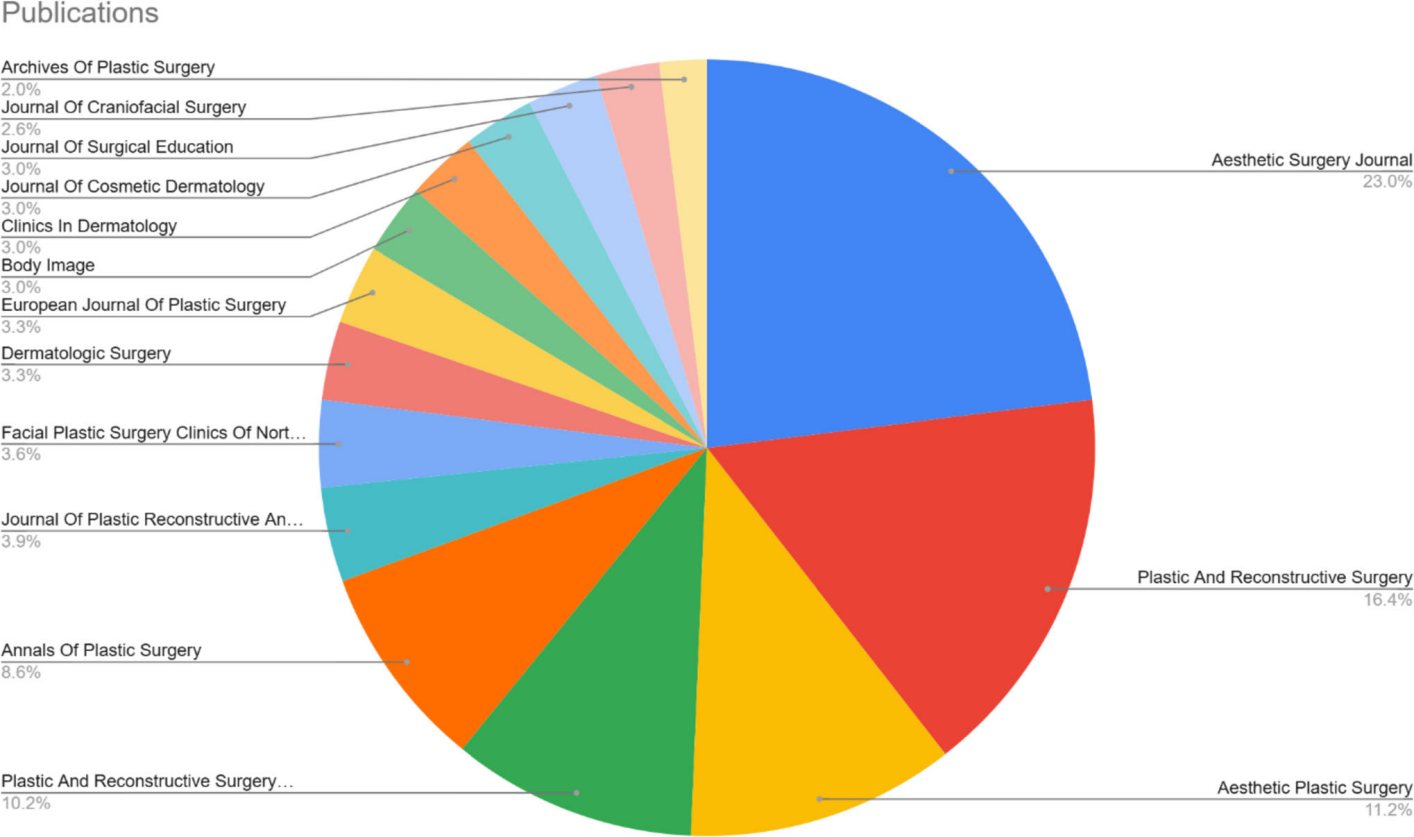
Most relevant journals

An analysis of publication sources indicates a concentrated distribution of research across a core group of specialized journals in aesthetic and plastic surgery, as shown in Fig. 3. The Aesthetic Surgery Journal is the leading publication venue, with 70 articles, followed by Plastic and Reconstructive Surgery with 50 publications. Aesthetic Plastic Surgery ranks third with 34 articles, while Plastic and Reconstructive Surgery Global Open and Annals of Plastic Surgery contribute 31 and 26 publications, respectively. A second tier of journals shows a moderate but noteworthy level of contribution. The Journal of Plastic Reconstructive and Aesthetic Surgery has published 12 articles, and Facial Plastic Surgery Clinics of North America has contributed 11. Several interdisciplinary journals also demonstrate engagement with the topic. Dermatologic Surgery and the European Journal of Plastic Surgery each published 10 articles, while Body Image, Clinics in Dermatology, the Journal of Cosmetic Dermatology, and the Journal of Surgical Education each contributed nine articles. Additionally, the Journal of Craniofacial Surgery and Archives of Plastic Surgery added eight and six publications, respectively. This distribution pattern highlights a strong concentration of literature within surgery-focused journals, while also reflecting the interdisciplinary nature of the field. The findings suggest that, although the core discourse is situated within the surgical community, related disciplines such as dermatology, psychology, and medical education are also actively contributing to the scholarly conversation.

### Country-Level Contributions

**Fig. 4:**
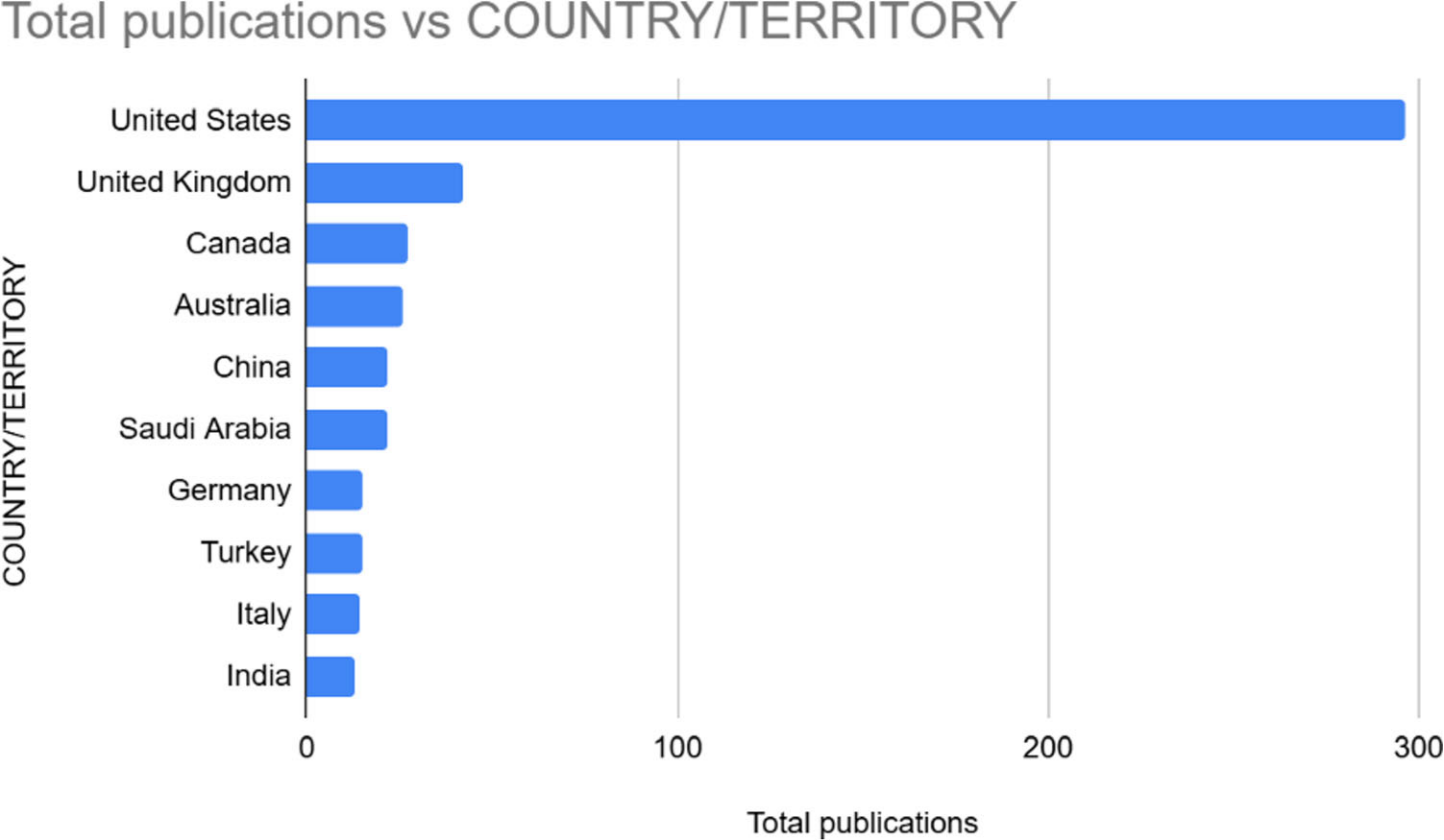
Top contributing countries

The analysis of country-level contributions reveals a highly uneven global distribution in research output on the intersection of social media and cosmetic surgery. As shown in Fig. 4, the USA is by far the most prolific contributor, accounting for the majority of publications in this domain, with close to 300 records. This dominance reflects the country's leading role in both aesthetic medicine and social media research. The United Kingdom follows at a significant distance, occupying the second position in terms of output. Other notable contributors include Canada, Australia, and China, each producing a moderate volume of research. Saudi Arabia, Germany, Turkey, Italy, and India also appear among the top ten publishing countries, although their output is comparatively lower. This geographic distribution highlights the concentration of academic activity in high-income, English-speaking countries, suggesting a potential imbalance in global research representation. The relatively limited contributions from regions such as Latin America, Africa, and Southeast Asia point to an opportunity for more inclusive and diversified research efforts in future studies.

### Most Influential Publications

The citation analysis of highly cited articles in social media and cosmetic surgery research reveals significant scholarly contributions that have shaped the field's development [19]. A seminal work in Aesthetic Plastic Surgery, with 171 citations, established a foundational understanding of how social media and online information influence aesthetic plastic surgery practice. This was followed by an influential study in Plastic and Reconstructive Surgery [26], garnering 144 citations, which examined the broader implications of social media on surgical practice. The temporal distribution of influential works demonstrates the field's evolution, with Branford et al. [4] study of #PlasticSurgery (136 citations) marking a significant advancement in understanding social media platforms' role in professional communication. Hanefeld et al. [14] work in Social Science and Medicine (129 citations) broadened the scope by examining medical tourism networks, while Sorice et al. [24] contributed valuable insights into patient-centered perspectives with 127 citations.

More recent contributions have addressed emerging aspects of social media influence [9]. Analysis of Instagram hashtags (124 citations) explored educational and marketing implications, while Farsi [12] comprehensive review (111 citations) examined healthcare providers' social media use. Walker et al. [27] study of social media's effect on young women's cosmetic surgery desires (107 citations) represents the field's expansion into psychological impacts. The publication pattern shows the concentration in prestigious surgical journals, particularly Plastic and Reconstructive Surgery and Aesthetic Surgery Journal, indicating the field's strong clinical foundation while encompassing broader societal implications. Table 1 illustrates the top listed articles included in the study.

**Table 1:** Top cited articles

Authors	Title	Source title	Cited by
Montemurro et al. [19]	The Influence of social media and Easily Accessible Online Information on the Aesthetic Plastic Surgery Practice: Literature Review and Our Own Experience	Aesthetic Plastic Surgery	171
Vardanian et al. [26]	Social media use and impact on plastic surgery practice	Plastic and Reconstructive Surgery	144
Branford et al. [4]	Plastic and Reconstructive Surgery	Plastic and Reconstructive Surgery	136
Hanefeld et al. [14]	Why do medical tourists travel to where they do? The role of networks in determining medical travel	Social Science and Medicine	129
Sorice et al. [24]	Social media and the plastic surgery patient	Plastic and Reconstructive Surgery	127
Dorfman et al. [9]	Plastic Surgery-Related Hashtag Utilization on Instagram: Implications for Education and Marketing	Aesthetic Surgery Journal	124
Farsi [12]	Social media and health care, part I: Literature review of social media use by health care providers	Journal of Medical Internet Research	111
Walker et al. [27]	Effects of social media use on desire for cosmetic surgery among young women	Current Psychology	107

### Co-Authorship of Authors Analysis

**Fig. 5:**
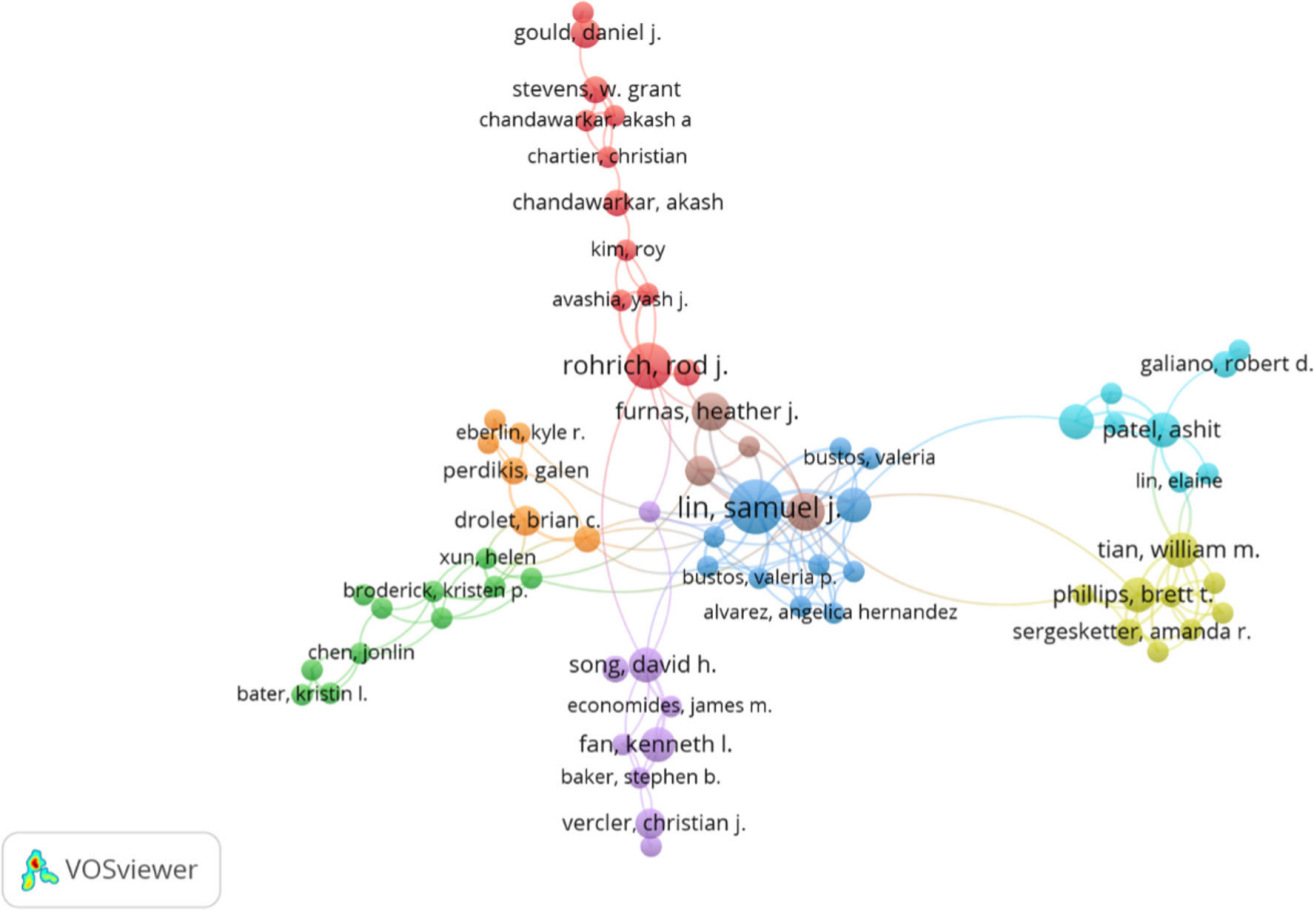
Co-authorship analysis of authors

The co-authorship analysis reveals intricate patterns of scholarly collaboration within the field of social media and cosmetic surgery research. As illustrated in Fig. 5, VOSviewer was employed to visualize collaborative networks among authors with at least two publications, reducing the original set of 1,868 authors to 209. This criterion enabled the identification of the most active and influential contributors based on both productivity and network centrality. Table 2 summarizes the top-ranked authors according to Total Link Strength (TLS), which reflects the cumulative strength of co-authorship connections, while the “Links” column indicates the number of distinct co-author relationships. The table also includes the number of documents published and total citations received, offering a holistic view of each author's academic impact. Samuel J. Lin stands out as the most interconnected researcher, with 19 collaborative links and the highest TLS (38). His influence is further supported by 13 publications and 283 citations, highlighting both extensive collaboration and scholarly productivity. Other prominent contributors include Bernard T. Lee and William M. Tian, with 14 and 10 links, respectively. Lee has authored six documents and received 100 citations, while Tian, with five publications and a TLS of 16, represents an emerging voice in the field.

**Table 2:** Collaboration network strength

Author	Links	Total link strength	Documents	Citations
Lin, Samuel J.	19	38	13	283
Lee, Bernard T.	14	20	6	100
Tian, William M.	10	16	5	2
Phillips, Brett T.	9	15	5	24
Patel, Ashit	7	12	5	14
Chen, Austin D.	5	11	4	100
Rohrich, Rod J.	8	11	9	296
Shiah, Eric	8	11	5	34
Song, David H.	8	11	5	264
Bustos, Valeria P.	9	10	2	0
Fan, Kenneth L.	5	10	5	108
Janis, Jeffrey E.	8	9	3	22
Sergesketter, Amanda R.	6	9	3	10
Valentine, Lauren	7	9	2	5
Weidman, Allan A.	7	9	2	5
Zeng, Steven L.	7	9	2	1
Alvarez, Angelica Hernandez	6	8	2	0
Foppiani, Jose	6	8	2	0
Furnas, Heather J.	6	8	6	213
Zhang, Gloria X.	7	8	2	8

Rod J. Rohrich presents a notable case of scholarly impact with 296 citations across nine publications, despite a relatively smaller collaborative network (eight links), suggesting a more focused but influential research agenda. Similarly, authors such as David H. Song (264 citations) and Heather J. Furnas (213 citations) demonstrate high citation counts within moderately sized collaborative clusters, indicating concentrated yet impactful scholarly contributions. In contrast, emerging scholars such as Bustos, Alvarez, and Foppiani display high levels of co-authorship activity but currently maintain lower citation counts, potentially reflecting recent entry into the research domain or growing visibility. These findings illustrate a diverse range of collaboration strategies within the field, from highly networked authors to those whose influence stems from more targeted scholarly outputs.

### Country-Level Collaboration Patterns

**Fig. 6:**
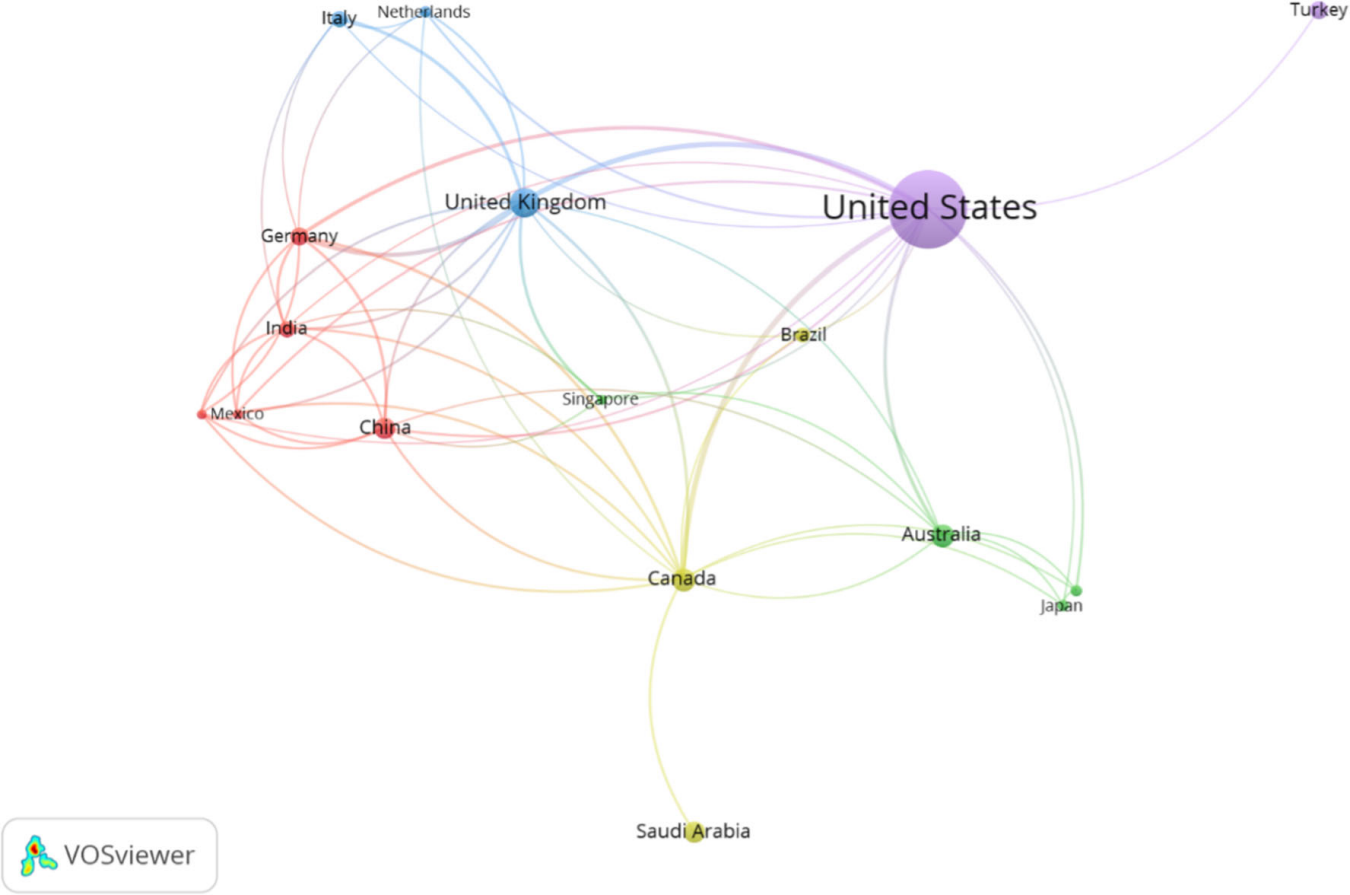
Collaboration patterns between the countries

The co-authorship analysis of international collaboration in social media and cosmetic surgery research reveals sophisticated patterns of global research partnerships (Fig. 6). The analysis reveals varying degrees of international cooperation shaped by factors such as research infrastructure, geographic proximity, and national academic priorities. The analysis, focused on countries meeting a threshold of 5 documents and zero citations, identified 17 key contributing nations from an initial pool of 115 countries, providing insights into core collaborative networks in this field. The USA demonstrates clear leadership in international research collaboration, evidenced by its extensive network metrics (16 links, 48 total link strength) and substantial research output (292 documents, 4,165 citations). This dominant position establishes the USA as a central hub for international research partnerships, facilitating connections across geographical regions and research communities. The United Kingdom emerges as a crucial intermediary in the global research network, particularly between North American and European research communities. With 13 links and 38 total link strength, coupled with significant research impact (43 documents, 798 citations), the UK's position reflects its important role in fostering trans-Atlantic research collaboration. This bridging function is further strengthened by Canada's strong international engagement (13 links, 31 total link strength), facilitating connections between North American and Asian research communities.

Asian nations demonstrate diverse collaboration patterns, with China and India showing active engagement (9 links each) despite moderate citation impacts. Saudi Arabia presents an interesting case, achieving substantial research impact (320 citations, 22 documents) with minimal international collaboration (1 link, 2 total link strength), suggesting effective domestic research programs. European contributions show varied patterns, with Germany demonstrating strong collaborative engagement (9 links, 26 total link strength) and significant impact (247 citations), while others maintain more focused collaboration networks. Table 3 below illustrates the collaboration patterns between the countries. This analysis reveals the complex interplay between geographical proximity, research capacity, and international collaboration in shaping the global research landscape. The patterns identified suggest opportunities for enhanced international collaboration, particularly among nations with established research programs but limited international engagement.

**Table 3:** The strength of the relationship between countries

Country	Links	Total link strength	Documents	Citations
Australia	7	10	26	556
Brazil	3	3	11	9
Canada	13	31	27	352
China	9	17	22	114
Germany	9	26	15	247
Greece	7	13	5	60
India	9	16	13	82
Italy	5	8	14	165
Japan	4	4	7	27
Mexico	7	14	6	43
Netherlands	5	7	7	123
Saudi Arabia	1	2	22	320
Singapore	5	7	6	128
South Korea	4	5	7	74
Turkey	1	1	15	105
United Kingdom	13	38	43	798
USA	16	48	292	4165

### Keyword Co-Occurrence and Thematic Evolution

The keyword co-occurrence analysis provides comprehensive insights into the thematic evolution and conceptual relationships within social media and cosmetic surgery research. The initial analysis identified 96 keywords from a pool of 1,044 that met the minimum occurrence threshold of three. After applying the thesaurus function in VOSviewer to consolidate related terms, 88 keywords remained, enabling a more focused examination of thematic patterns. The analysis reveals a clear hierarchical structure in keyword relationships, with "social media" emerging as the central concept (75 links, 288 total link strength, 128 occurrences). This dominance reflects its role as the foundational framework connecting various research aspects. "Plastic surgery" is the second most influential keyword (53 links, 161 total link strength). At the same time, "Instagram" demonstrates significant network strength (106) despite relatively fewer occurrences (33), indicating its crucial role in bridging different research domains.

**Fig. 7:**
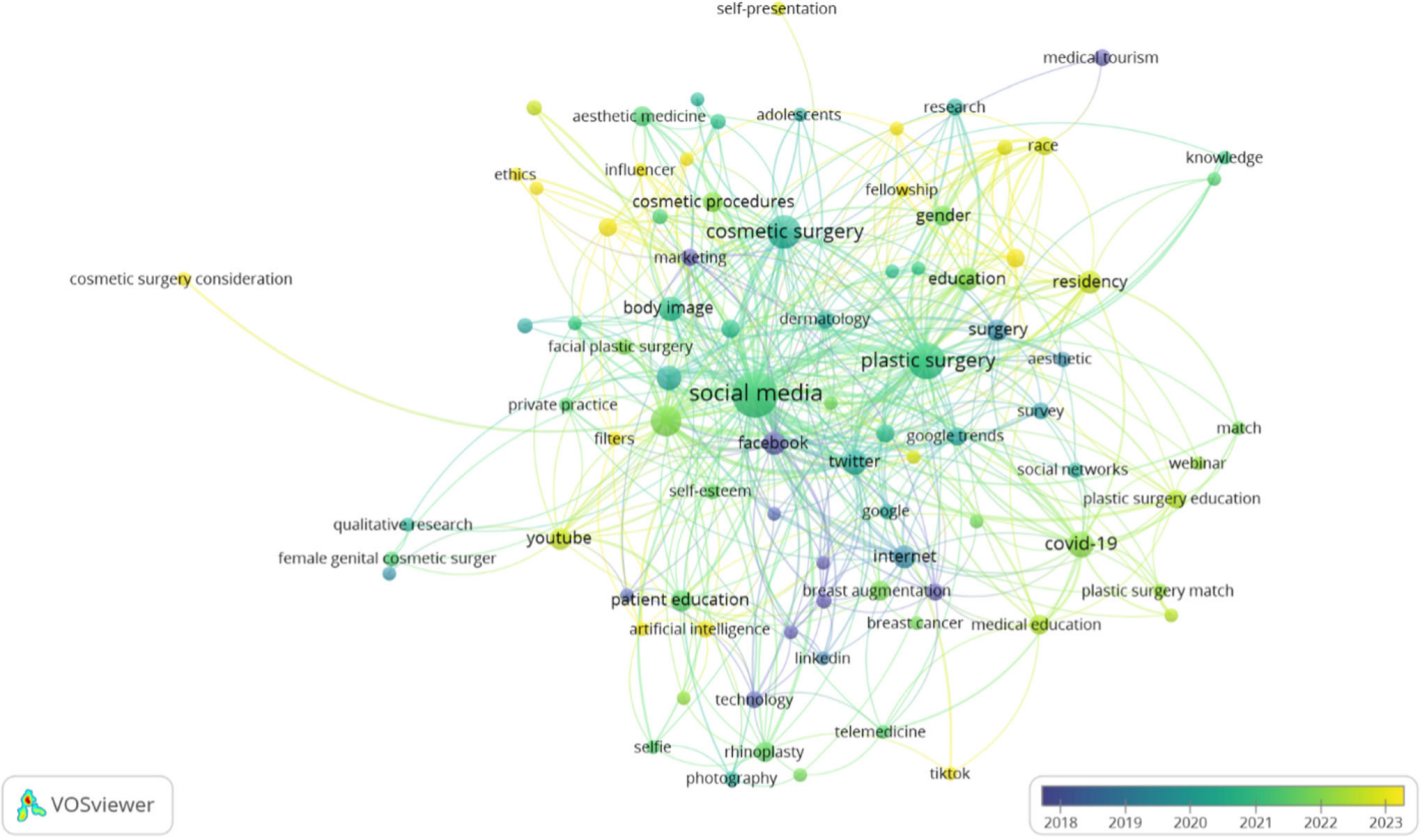
Network visualization of research topics in social media and plastic surgery (2018–2023)

Temporal analysis reveals distinct evolutionary patterns in research focus. Contemporary research frontiers are marked by keywords such as "diversity" (2023.143), "ethnicity" (2022.75), and "residency" (2022.5), while established concepts like "Facebook" (2018) and "surgery" (2019.3) represent the field's foundational elements. This temporal distribution demonstrates the field's progression from platform-specific investigations to broader societal and professional considerations. The network visualization identifies distinct thematic clusters, including educational themes (education, residency, plastic surgery education), technological developments (artificial intelligence, telemedicine), and clinical procedures (facial plastic surgery, rhinoplasty). These clusters, interconnected through high-strength keywords, illustrate the field's multifaceted nature and the complex relationships between different research domains. This structured analysis reveals both the current state of research focus and emerging trends in the field of social media and cosmetic surgery. Figure 7 illustrates the occurrence network below.

The network structure further reveals interesting patterns in research focus areas, demonstrated by clusters of related terms. For instance, educational themes (education, residency, plastic surgery education) form a distinct group. In contrast, technological aspects (artificial intelligence, telemedicine) and clinical procedures (facial plastic surgery, rhinoplasty) form their interconnected communities. Combined with quantitative metrics, this clustering provides a comprehensive understanding of how different research aspects in social media and cosmetic surgery interact and evolve. Table 4 shows the key topics and trends in social media and plastic surgery research (Based on weighted metrics and publication year)

**Table 4:** Key topics and trends in social media and plastic surgery research

Keyword	Links	Total link strength	Occurrences	Avg. pub. year
Social media	75	288	128	2021.125
Plastic surgery	53	161	64	2021
Instagram	40	106	33	2021.818
Cosmetic surgery	34	74	43	2020.372
Twitter	30	70	17	2020.118
Facebook	21	56	12	2018
Education	20	44	12	2021.833
Residency	20	41	12	2022.5
COVID-19	21	40	21	2021.905
Body image	17	36	16	2020.625
Internet	23	35	14	2019.214
Aesthetic surgery	18	32	15	2020.133
Diversity	15	28	7	2023.143
Race	14	27	6	2022.667
Gender	16	26	9	2021.667
YouTube	17	24	10	2022.5
Google Trends	16	22	6	2020
Surgery	14	22	10	2019.3
Cosmetic procedures	15	21	9	2021.889
Ethnicity	12	21	4	2022.75

The temporal analysis of keyword evolution in social media and cosmetic surgery research reveals significant shifts in research priorities and methodological approaches from 2018 to 2023. Recent keywords (2022-2023) demonstrate a clear transition toward social equity and educational considerations, evidenced by the emergence of terms such as "diversity" (2023.143), "ethnicity" (2022.75), and "race" (2022.667). This transformation indicates the field's progression beyond traditional clinical and technological frameworks to encompass broader societal implications. The field's maturation is reflected in its evolution from early platform-specific studies focused on Facebook and Twitter (2018-2019) to more sophisticated social implications and professional practice analyses. This development coincides with increased attention to cultural competency and inclusive practices, suggesting a growing recognition of social responsibility within aesthetic medicine. Simultaneously, the emergence of education-related keywords and technological terms like artificial intelligence and telemedicine indicates parallel advancement in professional training and service delivery methodologies. The analysis reveals an emerging research paradigm that integrates social responsibility, educational innovation, and technological advancement.

### Citation Analysis

**Fig. 8:**
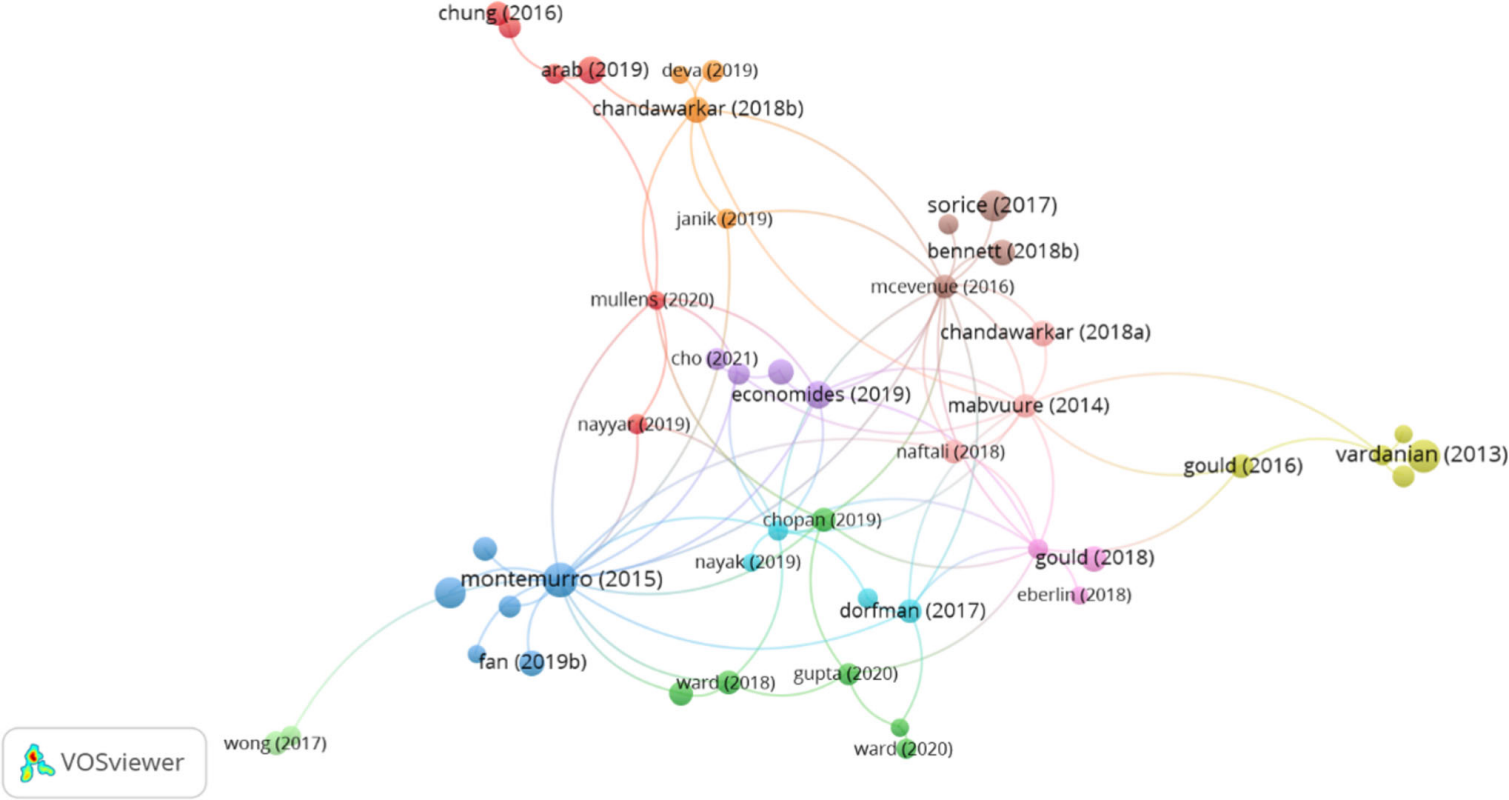
Co-authorship network of key researchers in social media and plastic surgery

The citation analysis uncovers key patterns of intellectual influence and thematic structuring within the intersection of social media and cosmetic surgery research. Through VOS viewer visualization, the analysis identifies key publications that have shaped the field's intellectual framework from 2013 to 2020, with node sizes reflecting citation impact and network connections demonstrating bibliographic relationships across research domains. Montemurro et al. [19] emerges as the field's cornerstone publication, garnering 171 citations and establishing 18 network links, thereby demonstrating its fundamental contribution to understanding the influence of social media on cosmetic surgery practice. This central position is complemented by Vardanian et al. [26] early influential work, which accumulated 144 citations despite limited network connections, indicating its pivotal role in establishing the field's theoretical foundations. The analysis further reveals significant contributions from works like Sorice et al. [24], with 127 citations, illustrating the field's continued development through diverse research approaches in Fig. 8.

The VOS viewer cluster analysis identifies distinct research communities, each represented by specific color coding. The blue cluster, anchored by Montemurro et al. [19], focuses on clinical practice implications, while the red cluster, including Arab et al. and Chung & Woo, examines patient behavior dynamics [1, 7]. The green cluster, featuring Chopan et al. [6], addresses professional education and training aspects, demonstrating the field's multifaceted nature. The temporal distribution analysis demonstrates the field's evolution from foundational works to contemporary contributions. While early publications like Vardanian et al. established core concepts, recent works such as Arab et al. and Economides et al., with 86 and 75 citations, respectively, indicate the field's continuing development [1, 10, 26]. This analysis, based on the top 20 most cited articles from 101 documents meeting the 20-citation threshold, reveals both the field's intellectual structure and its ongoing evolution through varied research perspectives and methodological approaches.

This comprehensive citation analysis thus provides crucial insights into the field's development, revealing how influential works have shaped research directions and contributed to the current understanding of social media's role in cosmetic surgery. The identified patterns of influence and collaboration offer valuable guidance for researchers and practitioners engaging in this dynamic field. Table 5 shows the highly cited articles in social media and cosmetic surgery considerations research.

**Table 5:** Highly cited articles in social media and cosmetic surgery considerations research

Article	URL	Links	Citations
Montemurro [19]	https://doi.org/10.1007/s00266-015-0454-3	18	171
Vardanian [26]	https://doi.org/10.1097/prs.0b013e318287a072	1	144
Sorice [24]	https://doi.org/10.1097/prs.0000000000003769	1	127
Arab [1]	https://doi.org/10.1097/gox.0000000000002333	2	86
Economides [10]	https://doi.org/10.1093/asj/sjy209	7	75
Gould (2018)	https://doi.org/10.1093/asj/sjx152	1	66
Chung [7]	https://doi.org/10.1111/ans.13393	1	52
Ward (2018)	https://doi.org/10.1007/s00266-017-1019-4	4	50
chopan (2019)	https://doi.org/10.1097/prs.0000000000005445	6	47

## Discussion

This bibliometric analysis offers a comprehensive overview of the scholarly landscape at the intersection of social media and cosmetic surgery, revealing critical patterns in publication trends, intellectual structure, and thematic evolution. Through quantitative synthesis and visual mapping, the findings illuminate how this interdisciplinary field has developed over the past decade and highlight several key insights.

The surge in publication output since 2015, particularly the exponential increase after 2020, signifies the field’s transition from an emerging niche to an established research area. This growth coincides with the global rise of visually oriented platforms such as Instagram and TikTok, and increasing societal normalization of aesthetic interventions. The sustained output over recent years suggests not only growing academic interest, but also a shift in public discourse and clinical attention toward the psychosocial dimensions of appearance enhancement. These trends align with prior claims that digital environments now play a central role in shaping beauty norms and body image dissatisfaction [21, 30].

The analysis of source journals reveals a clear dominance of surgical and aesthetic speciality outlets such as *Aesthetic Surgery Journal* and *Plastic and Reconstructive Surgery*, which collectively account for the majority of publications. This indicates that the core of this research remains anchored in clinical and procedural domains, despite its broader societal implications. However, the presence of interdisciplinary journals, such as *Body Image* and *Current Psychology*, reflects an expanding dialog between medical, psychological, and communication disciplines. Notably, highly cited works tend to be those that bridge theoretical constructs (e.g., social comparison, digital influence) with empirical observations, suggesting that impactful contributions are those that integrate both conceptual depth and real-world relevance. These works also frequently address clinical practice, patient decision-making, and ethical challenges, underscoring the field’s applied orientation.

The co-authorship and country collaboration analyses highlight an uneven distribution of research productivity and cooperation. The USA dominates both publication output and international collaborations, acting as a central hub in the global knowledge network. Canada, the United Kingdom, Australia, and Germany also display strong collaborative linkages, forming a high-capacity research cluster. Conversely, several emerging economies remain underrepresented in global partnerships. Countries like Turkey, Saudi Arabia, and India appear at the periphery of the collaboration network. This geographic imbalance raises concerns about epistemic centrality and calls for more inclusive knowledge production frameworks, particularly as cosmetic surgery and digital engagement grow rapidly in non-Western societies.

Keyword co-occurrence and temporal trend analyses indicate an evolution in research priorities. Early studies were largely platform-focused (e.g., Facebook, Twitter) and descriptive. Recent years have seen a marked shift toward more nuanced topics such as diversity, ethnicity, gender, and residency training. These changes reflect broader societal conversations around equity, representation, and professional education within aesthetic medicine. Simultaneously, the emergence of technological keywords, such as artificial intelligence and telemedicine, suggests a convergence between cosmetic surgery and digital innovation. These developments point to an emerging paradigm where surgical interventions are informed not only by clinical evidence but also by algorithmic influence, platform affordances, and mediated self-perception [6]. The integration of terms such as "body image," "gender," and "race" also reflects an important turn toward examining the ethical, psychological, and socio-cultural dimensions of cosmetic modification.

Citation network analysis highlights several foundational publications that have shaped the field’s conceptual scaffolding. Studies by Montemurro et al. and Vardanian et al. continue to serve as intellectual anchors, frequently cited for their exploration of social media’s clinical and behavioral implications [19, 26]. The clustering of citation networks into distinct communities, focusing on clinical practice, patient behavior, and medical education, demonstrates the field’s fragmentation into specialized yet interrelated streams. This structure reflects the multidisciplinary nature of the topic, but it may also signal a need for integrative frameworks that bridge clinical, psychological, and communication perspectives. While co-citation networks reveal robust intra-disciplinary dialogs, cross-disciplinary citations remain relatively limited. Promoting intellectual integration could enhance theoretical coherence and practical application.

The field appears well-positioned for continued development, with several clear directions for future research. These include the need for standardized approaches to social media integration in clinical practice, investigation of emerging ethical challenges, and enhancement of international collaboration networks. The strong foundation of existing research, combined with emerging focus areas addressing contemporary challenges, suggests a promising trajectory for continued evolution of this important field. This comprehensive analysis thus provides valuable insights for researchers and practitioners while highlighting critical areas for future investigation and development.

## Implications

The findings of this bibliometric analysis offer several important implications for research, practice, and policy at the intersection of social media and cosmetic surgery. As the field has grown rapidly over the past decade, this study provides a consolidated overview of its intellectual landscape and thematic progression, highlighting areas of influence, concentration, and emerging inquiry.

This study contributes to a clearer conceptual understanding of how digital media, particularly visual-based platforms like Instagram and TikTok, intersect with body image, beauty standards, and surgical enhancement. The thematic evolution from early concerns around platform effects to recent emphases on diversity, ethics, and education reflects a theoretical maturation in the field. Researchers are increasingly moving beyond simplistic exposure models to more nuanced frameworks, incorporating theories of social comparison, media literacy, and digital identity. The emergence of keywords related to race, gender, and inclusivity suggests growing scholarly interest in intersectional and socio-cultural approaches, which may guide future theoretical refinements.

The clustering of publications in specialized surgical journals highlights a methodological siloing within the field. While clinical perspectives dominate, there is an opportunity for greater interdisciplinary dialog with fields such as media studies, sociology, and public health. Furthermore, the citation analysis reveals a relatively small core of highly influential studies, suggesting that newer contributions may benefit from stronger engagement with foundational literature. This underscores the need for methodological pluralism, including mixed-methods approaches and longitudinal designs, to explore not only correlations but also causal mechanisms and lived experiences.

The dominance of plastic surgery–focused outlets and clinical themes underscores the practical relevance of social media in shaping patient expectations, motivations, and decision-making. Surgeons and aesthetic practitioners must remain critically aware of how algorithmic content, influencer culture, and user-generated images affect patients' perceptions of normalcy and ideal beauty. Educational initiatives should be developed to promote media literacy among both providers and patients, ensuring informed consent processes take into account the influence of digital appearance norms. Additionally, findings related to professional education and residency highlight the necessity of integrating social media ethics and digital professionalism into medical training programs.

## Limitations

While this study provides a systematic and data-driven overview of the scholarly landscape, several limitations must be acknowledged. First, the analysis relied solely on publications indexed in the Scopus and Web of Science databases, which may omit relevant works from regional journals or non-English publications. This language and indexing bias may result in an underrepresentation of perspectives from non-Western contexts, particularly from Asia, Latin America, and Africa, where social media usage patterns and cosmetic surgery trends may differ substantially. Second, bibliometric methods emphasize citation frequency and network structure but do not assess the content quality, methodological rigor, or theoretical contributions of individual articles. As a result, influential but critically debated works may receive the same weight as universally accepted contributions. This quantitative focus also limits the capacity to capture evolving nuances in research themes that may not yet have accumulated sufficient citations to be detected through network analysis. Third, the keyword co-occurrence analysis was dependent on author-supplied keywords, which can vary in precision and consistency. Although a thesaurus function was applied to mitigate terminological redundancy, some thematic fragmentation may remain. Additionally, VOSviewer visualizations, while effective for mapping structural relationships, cannot fully represent the causal, interpretive, or discursive complexity of the field.

## Future Directions

Building on the study's findings, several specific research directions warrant attention. First, future research should develop standardized bibliometric indicators to capture social media's dynamic influence on cosmetic surgery. This could include creating new metrics that account for both traditional academic impact and social media engagement levels, providing a more comprehensive understanding of research influence in this digital age. Second, there is a critical need for longitudinal bibliometric studies that track the evolution of research themes across different social media platforms. Such studies should incorporate emerging platforms and their unique influences on cosmetic surgery trends, moving beyond the traditional focus on established platforms like Facebook and Instagram. This approach would provide deeper insights into how different social media environments shape cosmetic surgery discourse and practice. Third, future research should address the geographical imbalance identified in the current analysis by developing targeted strategies for increasing international research collaboration. This could involve establishing multinational research networks specifically focused on studying social media's influence on cosmetic surgery across different cultural contexts, thereby enriching the global understanding of this phenomenon. Lastly, future bibliometric analyses should integrate alternative metrics that capture the impact of research beyond traditional academic citations, including social media mentions, professional forum discussions, and patient engagement metrics. This comprehensive approach would better reflect the full scope of influence that research has in both academic and practical settings within the cosmetic surgery field.
